# The Role of Surgery for Stage 0 Adenocarcinoma In Situ of the Lung: A National Analysis

**DOI:** 10.3390/jcm14176130

**Published:** 2025-08-29

**Authors:** Jessica Copeland, Eliza Neal, Tayyiaba Farooq, Endel Orav

**Affiliations:** 1 Department of Epidemiology, Harvard TH Chan School of Public Health, Boston, MA 02115, USA; 2 Department of Surgery, Massachusetts General Hospital, Boston, MA 02214, USA; 3 Rowan Virtua School of Osteopathic Medicine, Stratford, NJ 08084, USA; 4 Department of Biostatistics, Harvard TH Chan School of Public Health, Boston, MA 02115, USA

**Keywords:** stage 0 lung adenocarcinoma, ground glass nodules, surgical resection

## Abstract

**Objectives:** Overall survival (OS) of patients with stage 0 adenocarcinoma in situ (AIS) of the lung is not well characterized in the U.S. Specifically, there are a lack of data regarding the OS of patients with stage 0 AIS who do not receive treatment. We compared OS among stage 0 AIS patients who received surgery and those who received no treatment. **Methods:** OS of patients with stage 0 (TIS, N0, M0) AIS of the lung who received surgery versus no treatment from 2010 to 2018 in the National Cancer Data Base was evaluated using multivariable Cox proportional hazards modeling and propensity score-matched analysis. Predictors of surgery were evaluated using multivariable logistic regression. Survival outcomes based on surgical approach were evaluated in a propensity score-matched subgroup analysis. **Results:** Of the 897 patients who were diagnosed with stage 0 AIS, 716 (79.8%) underwent surgical resection. A propensity score-matched analysis of 134 patients who received no treatment and 134 patients who underwent surgery showed that the surgical group had a significantly improved OS at five-years 85.8% (95% CI: 74.2–92.4%) compared to the group who received no treatment 62.8% (95% CI: 50.1–72.7%) (log-rank, *p* < 0.0001). Subgroup propensity score-matched analysis showed no significant differences in OS at five-years in the surgical group consisting of 201 patients who underwent a wedge resection 90.8% (95% CI: 83.8–94.8) compared to 201 patients who underwent a lobectomy 94.9% (95% CI: 89.9–97.4%) (log-rank, *p* = 0.19). **Conclusions:** In this national analysis, stage 0 AIS patients who underwent surgery had significantly better OS when compared to patients who did not receive treatment.

## 1. Introduction

Adenocarcinoma is the most common subtype of non-small cell lung cancer (NSCLC) and represents approximately 40% of all NSCLC lung cancer cases [1]. Stage 0 disease, adenocarcinoma in situ (AIS), is characterized by lesions that are <3 cm with a non-invasive lepidic growth pattern and with ≤5 mm of minimally invasive adenocarcinoma [1,2,3]. It is typically detected on Computed Tomography (CT) imaging as a ground-glass nodule (GGN), tends to grow slowly, and is not immediately suggestive of malignancy. Accordingly, current guidelines for managing GGNs recommend routine follow-up with low-dose CT scans every 1–2 years for up to five years after initial detection, making surgical resection of GGNs an unnecessarily invasive treatment in many cases [3].

Still, stage 0 AIS of the lung has consistently been reported to achieve a 100% five-year overall survival (OS) rate following surgical resection, irrespective of surgical approach [4,5,6,7,8,9,10,11,12,13,14,15]. However, a vast majority of these findings stem from studies conducted in Asia, which limits their generalizability to populations in the United States. Further, in the last five years, only a few small studies from the United States [10], Europe [12], and Australia [14] have addressed OS outcomes for stage 0 AIS patients undergoing surgical resection. Moreover, there are no data to demonstrate that surgical intervention is needed to achieve satisfactory long-term survival rates. To address this gap in evidence, we aimed to identify the most effective treatment modality for stage 0 AIS of the lung in the United States by comparing the five-year OS rates of patients who underwent surgical treatment to those who received no treatment.

## 2. Materials and Methods

### 2.1. Ethics Statement

This study used publicly available, de-identified patient information from the National Cancer Database (NCDB). Therefore, our analysis is not considered to involve human subjects, constitute as human subject research, and is exempt from Institutional Review Board approval.

### 2.2. Data Source

For this study, we accessed data collected from the NCDB a data source managed by the American College of Surgeons (ACS), Commission on Cancer (CoC) and the American Cancer Society. The NCDB captures approximately 70% of all newly diagnosed lung cancer cases in the United States including Puerto Rico, gathering data from more than 1500 cancer centers and containing more than 30 million patient records [16]. Disease subtype and staging data are directly recorded in the NCDB using the American Joint Committee on Cancer 8th edition TNM classifications for our given study period. International Classification of Diseases for Oncology, 3rd edition (ICD-O-3) histology and topography codes were used for data extraction. Patients who underwent surgical resection were identified using surgical procedure of the primary site codes.

### 2.3. Study Design

Data were extracted from all patients diagnosed with stage 0 (TIS, N0, M0) AIS from 2010 to 2018. Patients were excluded if they received any neoadjuvant or adjuvant treatment, stereotactic body radiotherapy, had missing data, a prior cancer diagnosis, or a concomitant cancer diagnosis during the study period. Complete TNM staging according to the 8th addition was initially included in data analysis, but all patients were found to be N0 and M0 and thus only tumor size was kept in the final analysis. Patients were divided into two groups: patients who did not receive surgical treatment or any adjuvant therapy and those who underwent surgical resection and did not receive any adjuvant therapy.

The primary outcome was overall survival at five years, assessed from the time of diagnosis to the time of death or last known follow-up. Cox proportional hazards modeling and propensity score-matched analysis were used to evaluate OS across the two treatment groups. Covariates associated with patients undergoing surgery were evaluated using multivariable logistic regression. Survival outcomes based on surgical approach were evaluated in a propensity score-matched subgroup analysis that compared wedge resection against segmentectomy and wedge resection against lobectomy.

### 2.4. Statistical Analysis

**Figure 1:**
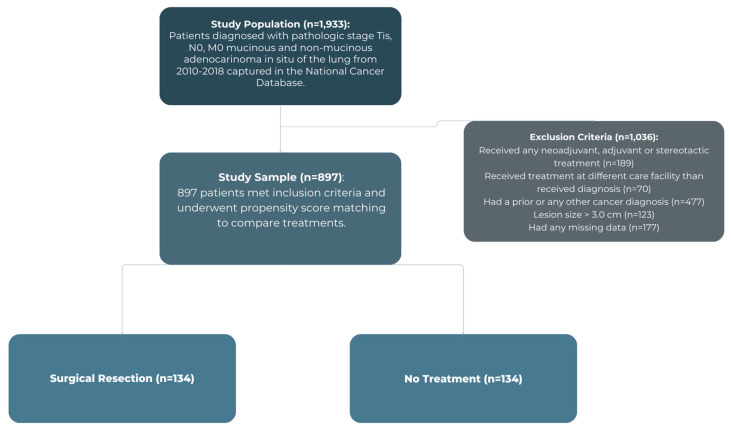
Study flow diagram.

Patients diagnosed with stage 0 (TIS, N0, M0) with lesions no greater than 3 cm and were classified as mucinous or non-mucinous adenocarcinoma in situ on histology from 2010 to 2018 were grouped based on treatment modality: surgical resection versus no treatment, as shown in Figure 1. Baseline characteristics and unadjusted outcomes were analyzed using Pearson’s chi-square test for categorical variables and Wilcoxon Rank Sum test for continuous variables. Covariates associated with surgical treatment were assessed using a multivariable logistic regression model that included: age, sex, race, insurance status, income, education, facility type, distance from facility, year of diagnosis, Charlson–Deyo Comorbidity (CDCC) score, and tumor size. An adjusted multivariable Cox proportional hazards model was then used to assess differences in overall survival between the two groups.

An adjusted propensity score-matched analysis of those undergoing surgical resection and those not receiving surgery was then performed. Propensity scores were developed, defined as the probability of patients who did not receive any treatment and those who underwent surgical resection for stage 0 AIS, conditional on sociodemographic and prognostic variables. Covariates in our propensity score-matched analysis included: age, sex, race, insurance status, income, education, facility type, distance from facility, year of diagnosis, CDCC score, and tumor size. Covariates were determined a priori to be clinically relevant. Propensity score-matching was used over inverse probability weighting to better estimate the average treatment effect for patients.

Using a greedy 1:1 matching scheme without replacement and a radius-matching caliper of 0.01 the most appropriately matched pairs were identified. After matching, balance was assessed using absolute standardized differences. Following propensity-score matching, Kaplan–Meier analysis was used to assess overall long-term survival of the two groups. Overall survival was measured from the time of diagnosis to the time of death or last known follow-up. This model was then applied to a secondary subgroup analysis that assessed overall survival within the surgical cohort across different surgical approaches (wedge resection versus segmentectomy and wedge resection versus lobectomy). Model balance and diagnostics were evaluated with no violation of major assumptions identified. For all comparisons, a two-sided *p*-value of 0.05 was used to define statistical significance. Statistical analysis was performed using Stata/MP software, version 13.1 for Mac (StataCorp, College Station, TX, USA).

## 3. Results

### 3.1. Predictors of Surgical Intervention and Overall Survival

A total of 716 (79.8%) patients of 897 patients who met study inclusion criteria underwent surgical resection for stage 0 AIS from 2010 to 2018. Baseline patient characteristics by treatment modality are represented in Table 1. In univariate analysis, patients undergoing surgical resection were more likely to be younger, female, and have smaller tumors. No significant differences were seen across race, income, education, mean distance from the treating facility, or comorbidity score.

Multivariable analysis evaluating independent predictors of patients with stage 0 AIS to receive surgery are presented in Table 2. Females and younger patients were more likely to receive surgery and those with larger tumors were less likely to receive surgery. Factors associated with survival among patients with stage 0 AIS who received surgery are also shown in Table 2. Decreased age and female sex were associated with a survival benefit, while black race was associated with an increased risk of mortality compared to white race. Among the entire cohort with stage 0 AIS multivariable Cox proportional hazards analysis revealed that surgery was associated with improved overall survival (0.25; CI: 0.16–0.40, *p* < 0.001).

### 3.2. Propensity Score-Matched Analysis

Propensity score matching was performed to create two groups of 134 patients diagnosed with AIS who either underwent surgical resection or did not receive surgery. Both groups were well-balanced after propensity score matching as shown in Table 3. All absolute standardized mean differences were less than or equal to 10.2.

**Figure 2:**
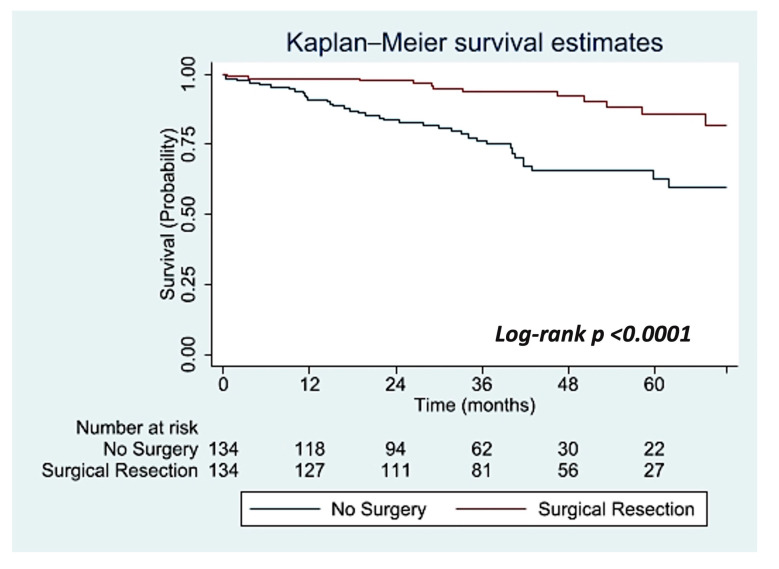
Propensity score-matched overall survival of stage 0 AIS patients by treatment group. Confidence intervals were set at 95% and are represented by color, *blue* (no surgery) and *red* (surgical resection).

Overall survival was significantly improved at 24, 36, 48, and 60 months in the surgical group compared to the no treatment group. OS at 24 months in the surgical group was 97.7% (95% CI: 92.9–99.2%) compared to the no treatment group at 83.6% (95% CI: 75.9–88.9%). OS at 36 months in the surgical group was 93.8% (95% CI: 87.3–97.0%) compared to the no treatment group at 76.3% (95% CI: 67.3–83.1%). OS at 48 months in the surgical group was 92.2% (95% CI: 84.6–96.1%) compared to the no treatment group at 65.7% (95% CI:54.7–74.6%). Most notably, overall survival at five years was significantly improved in the surgical group at 85.8% (95% CI: 74.2–92.4%) compared to the no treatment group at 62.8% (95% CI: 50.1–72.7%) (log-rank, *p* < 0.0001) as is illustrated in Figure 2.

**Figure 3:**
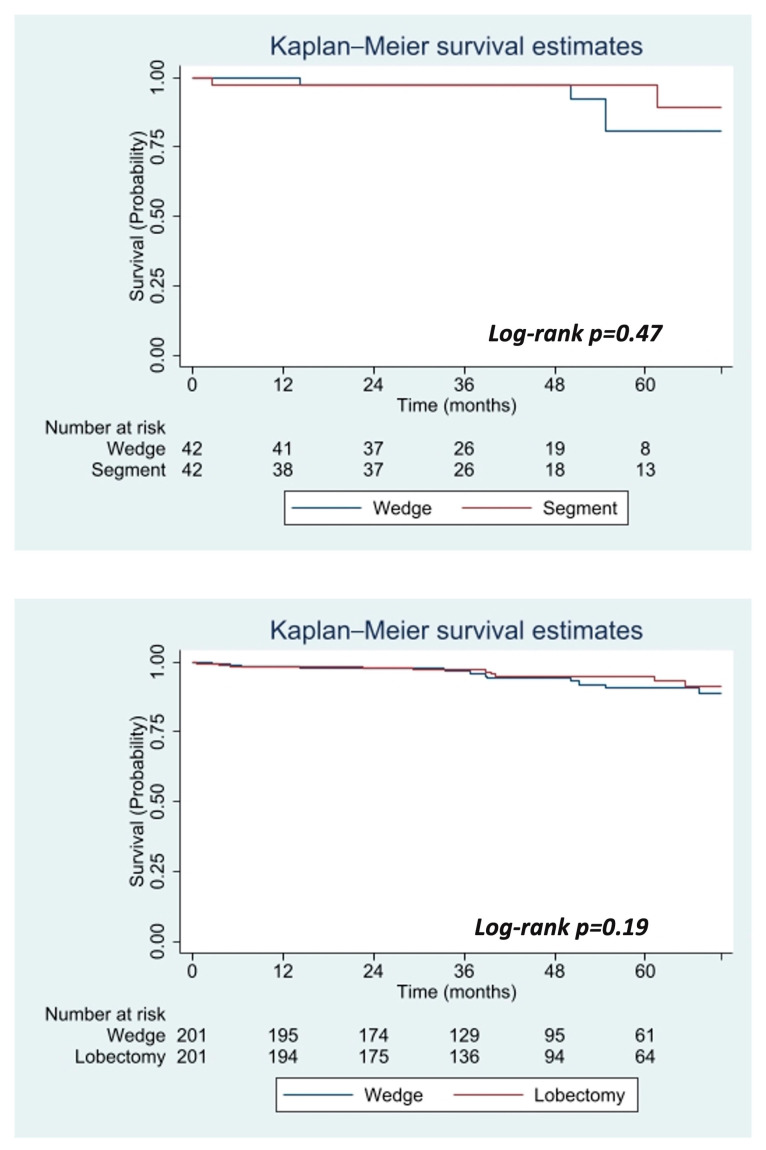
Propensity score-matched overall survival of stage 0 AIS surgical patients by surgical approach. Confidence intervals were set at 95% and are represented by color, *blue* (Wedge) and *red* (Segment) for the (**top**) image and *blue* (Wedge) and *red* (Lobectomy) for the (**bottom**) image.

Two additional propensity score-matched analyses were performed among stage 0 AIS patients who received surgery to detect a survival benefit across different surgical approaches as demonstrated in Figure 3. In this subgroup analysis, patients who underwent a wedge resection were compared to those who received a segmentectomy and against those who underwent a lobectomy. Among two groups of 42 patients with stage 0 AIS who underwent surgical resection there was no significant difference in OS at five years between those who underwent a wedge resection 80.1% (95% CI: 54.7–92.6%) compared to a segmentectomy 97.6% (95% CI: 83.9–99.7%) (log-rank, *p* = 0.47). Furthermore, among two groups of 201 stage 0 AIS patients who underwent surgical resection there was no significant difference in OS at five-years between those who underwent a wedge resection 90.8% (95% CI: 83.8–94.8) compared to a lobectomy 94.9% (95% CI: 89.9–97.4%) (log-rank, *p* = 0.19).

## 4. Discussion

This study compared OS among patients with stage 0 AIS of the lung who received surgical treatment to those who received no treatment and found that OS was significantly improved at 24, 36, 48, and 60 months in the group who received surgery. No significant differences in OS were found across varying surgical approaches: wedge resection versus segmentectomy and wedge resection versus lobectomy for stage 0 AIS of the lung. Further, comorbidity score was not found to be a significant predictor of OS for patients who underwent surgery.

These findings suggest that surgical resection improves OS in stage 0 AIS of the lung and are consistent with other international studies that show a survival benefit associated surgical resection for stage 0 AIS, regardless of surgical approach [4,5,7,12,14]. This study contributes to the existing literature by demonstrating a survival benefit among patients who undergo surgical resection when directly compared to patients who were surveilled and received no treatment.

There are several notable limitations of this study. While we aimed to reduce bias and confounding by performing a multivariable prediction model for surgical intervention and survival, this study is retrospective and still has the potential for confounding and selection bias. Since the non-treatment group did not undergo pathologic confirmation, these data may overestimate the overall survival benefit of those treated surgically. There is also a possibility that patients in the nonsurgical group had missed invasive disease and were incorrectly staged with AIS of the lung. That is, patients who underwent surgery likely had a more comprehensive pathologic evaluation of their tumor compared to those who did not receive surgery and there is a higher potential for nonsurgical patients to have missed invasive disease. However, we attempted to reduce this likelihood by excluding all patients who received any cancer diagnosis during the period of the study, as it would be unlikely that nonsurgical patients diagnosed with stage 0 AIS of the lung would have an unrecognized invasive component or missed upstaging long-term.

Additionally, it is important to recognize that nonsurgical patients with stage 0 AIS of the lung may be underreported in the NCDB, especially in cases where patients are diagnosed by interventional pulmonologists rather than surgeons, as the NCDB is primarily directed for use among surgeons. Other limitations of the NCDB include lack of data on pulmonary function testing, smoking, frailty, cancer recurrence, and the use of a comorbidity index rather than individual comorbidities.

This study was also limited by small sample sizes as this clinical situation, while becoming more common, is still fairly unique. This is especially notable for the surgical subgroups that underwent segmentectomy versus wedge resection for stage 0 AIS, which showed no difference in treatment modalities. However, we note that a difference may be appreciated if analyzed under a larger sample size. The surgical subgroup analysis comparing those that underwent lobectomy versus wedge resection was adequately powered to reach a confidence level of 95% with the real value falling within 5% of the measured value. Still, further research is needed to better appreciate the appropriateness of individual surgical interventions for stage 0 AIS, as lobectomy may cause increased morbidity and impact short-term perioperative outcomes not evaluated in this study. Further, the follow-up period in this study was relatively short for an indolent disease and may not fully capture long-term survival trends. Additional studies with longer follow-up periods would add to the evidence presented in this study. Lastly, overall generalizability of the study may be limited as these data primarily reflect white populations who received care at academic or comprehensive cancer facilities.

Nevertheless, this study is the first of its kind to shed light on survival outcomes based on treatment modality for Stage 0 AIS of the lung in a United States population. Our findings support long-term survival benefits for patients with Stage 0 AIS of the lung who undergo surgical resection compared to those who do not receive treatment. These results also demonstrate the ability to achieve long-term survival benefits by performing a wedge resection when compared to more aggressive operations such as a lobectomy for these patients.

## 5. Conclusions

In this national analysis of patients with stage 0 AIS of the lung, surgery was associated with improved OS at 24, 36, 48, and 60 months compared to those who received no treatment. While surgical treatment for stage 0 AIS has been increasing, it is still not a widely adopted practice. This study supports surgeons performing surgical resections for stage 0 AIS patients of the lung and suggests that a wedge resection achieves similar long-term survival benefits when compared to a lobectomy among this population.

## Figures and Tables

**Table 1:** Baseline characteristics of stage 0 AIS patients by treatment group.

*Predictors*		Non-Treatment Group	Surgical Group	*p*-Value
		181	716	
*Sex*	Male	73 (40.3%)	180 (25.1%)	<0.001
	Female	108 (59.7%)	536 (74.9%)	
*Age, mean years (SD)*		72.8 (9.7)	66.3 (9.7)	<0.001
*Race, n (%)*	White	155 (85.6%)	597 (83.4%)	0.86
	Black	13 (7.2%)	62 (8.7%)	
	Asian	10 (5.5%)	46 (6.4%)	
	Other	3 (1.7%)	9 (1.3%)	
*Insurance Status n (%)*	Uninsured	2 (1.1%)	10 (1.4%)	0.002
	Private	33 (18.2%)	235 (32.8%)	
	Medicaid	7 (3.9%)	30 (4.2%)	
	Medicare	132 (72.9%)	431 (60.2%)	
	Other	7 (3.9%)	10 (1.4%)	
*Annual Income n (%)*	<USD 40,227	22 (13.9%)	80 (12.6%)	0.55
	USD 40,227–50,353	33 (20.9%)	116 (18.3%)	
	USD 50,354–63,332	39 (24.7%)	140 (22.1%)	
	>USD 63,332	64 (40.5%)	297 (46.9%)	
*Education Percentage Without High School Diploma, n (%)*	>17.6%	26 (16.3%)	105 (16.6%)	0.15
	10.9–17.5%	43 (26.9%)	143 (22.6%)	
	6.3–10.8%	52 (32.5%)	175 (27.6%)	
	<6.3%	39 (24.4%)	211 (33.3%)	
*Facility Type, n (%)*	Community Cancer Facility	7 (3.9%)	24 (3.4%)	0.024
	Comprehensive Cancer Facility	84 (46.7%)	247 (34.8%)	
	Academic/Research Facility	57 (31.7%)	291 (41.0%)	
	Integrated Network Facility	32 (17.8%)	148 (20.8%)	
*Distance from Facility, mean miles (SD)*		30.1 (107.9)	52.6 (237.6)	0.24
*Year of Diagnosis, n (%)*	2010	1 (0.6%)	4 (0.6%)	0.064
	2011	2 (1.1%)	7 (1.0%)	
	2012	7 (3.9%)	39 (5.4%)	
	2013	12 (6.6%)	53 (7.4%)	
	2014	12 (6.6%)	97 (13.5%)	
	2015	21 (11.6%)	120 (16.8%)	
	2016	31 (17.1%)	105 (14.7%)	
	2017	48 (26.5%)	158 (22.1%)	
	2018	47 (26.0%)	133 (18.6%)	
*CDCC Score, n (%)*	0	93 (51.4%)	419 (58.5%)	0.16
	1	54 (29.8%)	205 (28.6%)	
	2	25 (13.8%)	64 (8.9%)	
	3	9 (5.0%)	28 (3.9%)	
*Tumor Size, mean size in mm (SD)*		16.7 (6.8)	13.1 (6.2)	<0.001

**Table 2:** Predictors of surgical intervention and overall survival in stage 0 AIS patients.

*Predictors*		Odds Ratio (95% CI, *p*-Value)	Hazards Ratio (95% CI, *p*-Value)
*Surgery* *(reference no treatment)*		NA	0.25 (0.16–0.40, <0.001)
*Sex (male vs. female)*		1.86 (1.23–2.80, 0.003)	0.55 (0.36–0.84, 0.005)
*Age (per year)*		0.92 (0.90–0.95, <0.001)	1.05 (1.02–1.08, 0.001)
*Race* *(reference white)*	Black	1.52 (0.68–3.38, 0.31)	2.08 (1.08–3.98, 0.03)
	Asian	1.10 (0.50–2.46, 0.80)	0.52 (0.15–1.78, 0.30)
	Other	0.74 (0.16–3.55, 0.71)	2.89 (0.16–52.45, 0.47)
*Insurance status (reference uninsured)*	Private	2.38 (0.41–13.73, 0.33)	0.99 (0.12–8.08, 0.99)
	Medicaid	1.33 (0.19–9.36, 0.78)	1.31 (0.13–12.78, 0.82)
	Medicare	3.3 (0.57–19.44, 0.18)	1.07 (0.13–8.78, 0.06)
	Other	1.75 (0.19–16.44, 0.63)	2.01 (0.17–24.02, 0.58)
*Annual income* *(reference < 40,227)*	USD 40,227–50,353	1.38 (0.68–2.81, 0.38)	0.55 (0.27–1.14, 0.11)
	USD 50,354–63,332	1.64 (0.79–3.42, 0.19)	0.68 (0.34–1.38, 0.29)
	>USD 63,332	1.68 (0.78–3.60, 0.18)	0.73 (0.35–1.50, 0.39)
*Education percentage without high school diploma (reference < 17.6%)*	10.9–17.5%	0.56 (0.30–1.07, 0.08)	0.91 (0.46–1.79, 0.79)
	6.3–10.8%	0.67 (0.34–1.31, 0.24)	1.03 (0.51–2.08, 0.93)
	<6.3%	0.92 (0.43–1.98, 0.83)	1.04 (0.47–2.32, 0.91)
*Facility type* *(reference community cancer facility)*	Comprehensive Cancer Facility	0.56 (0.20–1.56, 0.27)	0.75 (0.26–2.18, 0.60)
	Academic/Research Facility	0.71 (0.25–1.99, 0.51)	0.62 (0.21–1.84, 0.39)
	Integrated Network Facility	0.76 (0.25–2.28, 0.63)	0.97 (0.32–2.96, 0.96)
*Distance from facility (per mile)*		1.00 (0.99–1.01, 0.52)	1.01 (0.99–1.01, 0.55)
*Year of diagnosis* *(per year)*		0.84 (0.75–0.94, 0.003)	1.05 (0.93–1.20, 0.42)
*CDCC score* *(reference 0)*	1	0.90 (0.57–1.42, 0.65)	1.35 (0.83–2.19, 0.22)
	2	0.69 (0.38–1.27, 0.24)	2.18 (0.25–3.81, 0.06)
	3	0.90 (0.38–2.21, 0.84)	1.21 (0.48–3.08, 0.69)
*Tumor size (per mm)*		0.94 (0.91–0.97, <0.001)	1.00 (0.97–1.03, 0.95)

**Table 3:** Propensity score-matched analysis of stage 0 AIS patients by treatment group.

*Predictors*		Non-Treatment Group	Surgical Group	Absolute Standardized Difference (%)
		134	134	
***Female Sex, n (%)***		85 (63.4%)	82 (61.2%)	4.8
***Age, mean years (SD)***		71.8 (9.5)	72.2 (7.8)	3.9
***Race, n (%)***	White	112 (83.6%)	107 (79.9%)	6.1
	Black	10 (7.5%)	13 (9.7%)	8.3
	Asian	10 (7.5%)	11 (8.2%)	3.1
	Other	2 (1.5%)	3 (2.2%)	6.2
***Insurance Status, n (%)***	Uninsured	1 (0.7%)	4 (3.0%)	2.6
	Private	29 (21.6%)	26 (19.4%)	5.2
	Medicaid	4 (3.0%)	4 (3.0%)	<0.1
	Medicare	96 (71.6%)	97 (72.4%)	1.6
	Other	4 (3.0%)	3 (2.2%)	<0.1
***Annual Income, n (%)***	<USD 40,227	18 (13.4%)	20 (14.9%)	4.6
	USD 40,227–50,353	27 (20.1%)	30 (22.4%)	5.6
	USD 50,354–63,332	33 (24.6%)	34 (25.4%)	1.8
	>USD 63,332	56 (41.8%)	50 (37.3%)	9.1
***Education Percentage Without High School Diploma, n (%)***	>17.6%	24 (17.9%)	26 (19.4%)	4.2
	10.9–17.5%	32 (23.9%)	30 (22.4%)	3.5
	6.3–10.8%	42 (31.3%)	50 (37.3%)	11
	<6.3%	36 (26.9%)	28 (20.9%)	10.2
***Facility Type, n (%)***	Community Cancer Facility	5 (3.7%)	3 (2.2%)	8.4
	Comprehensive Cancer Facility	62 (46.3%)	59 (44.0%)	4.6
	Academic/Research Facility	46 (34.3%)	49 (36.6%)	4.7
	Integrated Network Facility	21 (15.7%)	23 (17.2%)	3.8
***Distance from Facility, mean miles (SD)***		31.1 (116.7)	36.4 (201.4)	2.8
***CDCC Score, n (%)***	0	69 (51.5%)	69 (51.5%)	<0.1
	1	38 (28.4%)	37 (27.6%)	1.6
	2	18 (13.4%)	19 (14.2%)	2.3
	3	9 (6.7%)	9 (6.7%)	<0.1
***Year of Diagnosis, (IQR)***		2014 (2011, 2017)	2014 (2011, 2017)	9.4
***Tumor Size, mm (SD)***		16.1 (6.8)	16.6 (7.2)	6.7

## Data Availability

The data underlying this article are available from the National Cancer Database. The datasets were derived from sources in the public domain using deidentified Participant User Data Profiles that are compliant with the Health Insurance and Portability and Accountability Act and submitted to the Commission on Cancer’s National Cancer Database and can be found at: https://www.facs.org/quality-programs/cancer-programs/national-cancer-database/.

## Abbreviations

The following abbreviations are used in this manuscript:

ACS	American College of Surgeons
AIS	Adenocarcinoma in Situ
CDCC	Charlson–Deyo Comorbidity
CoC	Commission on Cancer
CT	Computed Tomography
GGN	Ground-Glass Nodule
ICD-O-3	International Classification of Diseases for Oncology, 3rd edition
NCDB	National Cancer Database
NSCLC	Non-Small Cell Lung Cancer
OS	Overall Survival
